# The complete mitogenome of *Eriocrania semipurpurella alpina* Xu, 1990 (Lepidoptera: Eriocraniidae)

**DOI:** 10.1080/23802359.2025.2609369

**Published:** 2025-12-30

**Authors:** Xiaoning Zhang, Wenbo Feng, Qingbai Hou

**Affiliations:** ^a^ College of Life Sciences, Qinghai Normal University, Xining, PR China; ^b^ Academy of Plateau Science and Sustainability, Qinghai Normal University, Xining, PR China; ^c^ Qilian Mountain Southern Slope Forest Ecosystem Research Station, Huzhu County, Qinghai Province, PR China

**Keywords:** Mitogenome, Eriocraniidae, *Eriocrania semipurpurella alpina*, leaf miner

## Abstract

*Eriocrania semipurpurella alpina* Xu, 1990 is a leaf miner of birch trees in Qinghai Province, China. The complete mitogenome of *E. semipurpurella alpina* was reported in this study. The mitogenome contains 15,107 base pairs. Its nucleotide composition is 40.5% A, 8.1% G, 40.1% T, and 11.3% C. The genome encodes a set of 37 genes, including 13 protein-coding genes (PCGs), 22 tRNA genes, 2 rRNA genes, and one control region. Phylogenetic analysis supports the monophyly and primitive origin of Eriocraniidae.

## Introduction

Eriocraniidae are one of the most primitive small families of Lepidoptera, widespread in the Holarctic. This family comprises approximately 29 species classified into five genera (Van Nieukerken et al. 2011). Two species and one subspecies have been reported to be distributed in China: *E. sparrmannella*, *E. semipurpurella* Stephen, and *E. semipurpurella alpina* (Yang 1977; Xu et al. 1990). Eriocraniid moths are small day-flying moths, which have rudimentary mandibles and a short proboscis, with each mesotibia bearing a single spur. Their wing venation is complete and similar between the forewings and hindwings. They are among the first insects to emerge in spring, often preceding the activity of many other species (Powell 2009). *Eriocrania. semipurpurella alpina* is a leaf miner of birch trees, *Betula* spp., in Qinghai Province, China. The adults fly during the daytime and lay eggs on the leaf buds; their larvae feed on the leaves in a mine. *E. semipurpurella alpina* completes one generation per year in Qinghai. Their eggs hatched before the leaves of the trees completely formed, hardened, and produced repellent tannins. The larvae feed between the epidermal layers, maturing in two weeks. Once mature, they drop to the ground and spin a cocoon in the soil where they overwinter. Parasitoids play a significant role in controlling *E. semipurpurella alpina* populations in nature (Li et al. 2016).

## Materials and methods

**Figure 1.**
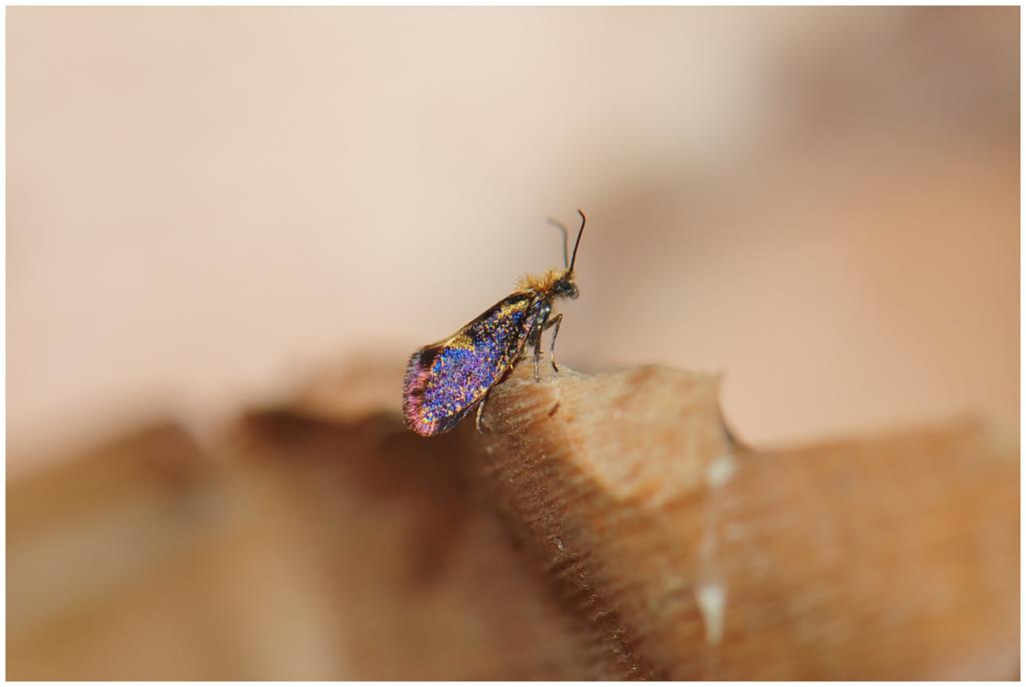
The studied specimen of *Eriocrania semipurpurella alpina* (voucher number: QNU2023L000161). Photograph by Qingbai Hou.

The adult specimens of *E. semipurpurella alpina* (Figure 1) were collected in May 2023 from Huzhu County, Qinghai Province, China (N36°51′44″, E102°34′20″). The specimens were preserved in absolute ethanol and stored at −20 °C in the Insect Collection, Qinghai Normal University, Xining, China (Qingbai Hou, mailto: bleding@126.com) under the voucher number QNU2023L000161. The total genomic DNA was extracted from the thorax muscles of five individuals using the TIANGEN Genomic DNA Extraction Kit (TIANGEN, Beijing, China) according to the manufacturer’s instructions. Then, Genomic DNA was used for Next Generation Sequencing. Briefly, DNA was fragmented by sonication. A 350-bp fragments library was constructed using the NEB Next Ultra™ DNA Library Prep Kit for Illumina and sequenced on an Illumina NovaSeq 6000 platform (Novogene Bioinformatics Institute, Tianjin, China) using paired-end 150 bp methods. The reads were assembled with SPAdes v3.14.1 (Bankevich et al. 2012) and MitoZ v3.6 (Meng et al. 2019). Then, the assembled mitogenome was annotated using the MITOS Web Server (Bernt et al. 2013) and manually confirmed by comparing with other Lepidoptera species. The phylogenetic relationship was inferred based on the 13-protein-coding genes from 24 Lepidoptera species and 2 Trichoptera outgroup species using MrBayes v.3.2.7a (Ronquist et al. 2012) with Bayesian inference (BI) methods (Figure 3). The BI analysis was conducted with the Markov chain Monte Carlo analysis run for 10,000,000 generations, sampled every 1000th generation and with a burn-in of 25%. Bayesian posterior probabilities (PP) >0.95 were interpreted as strongly supported. Finally, phylogenetic trees were visualized using FigTree v.1.4.4 software (Rambaut 2018).

## Results

**Figure 2.**
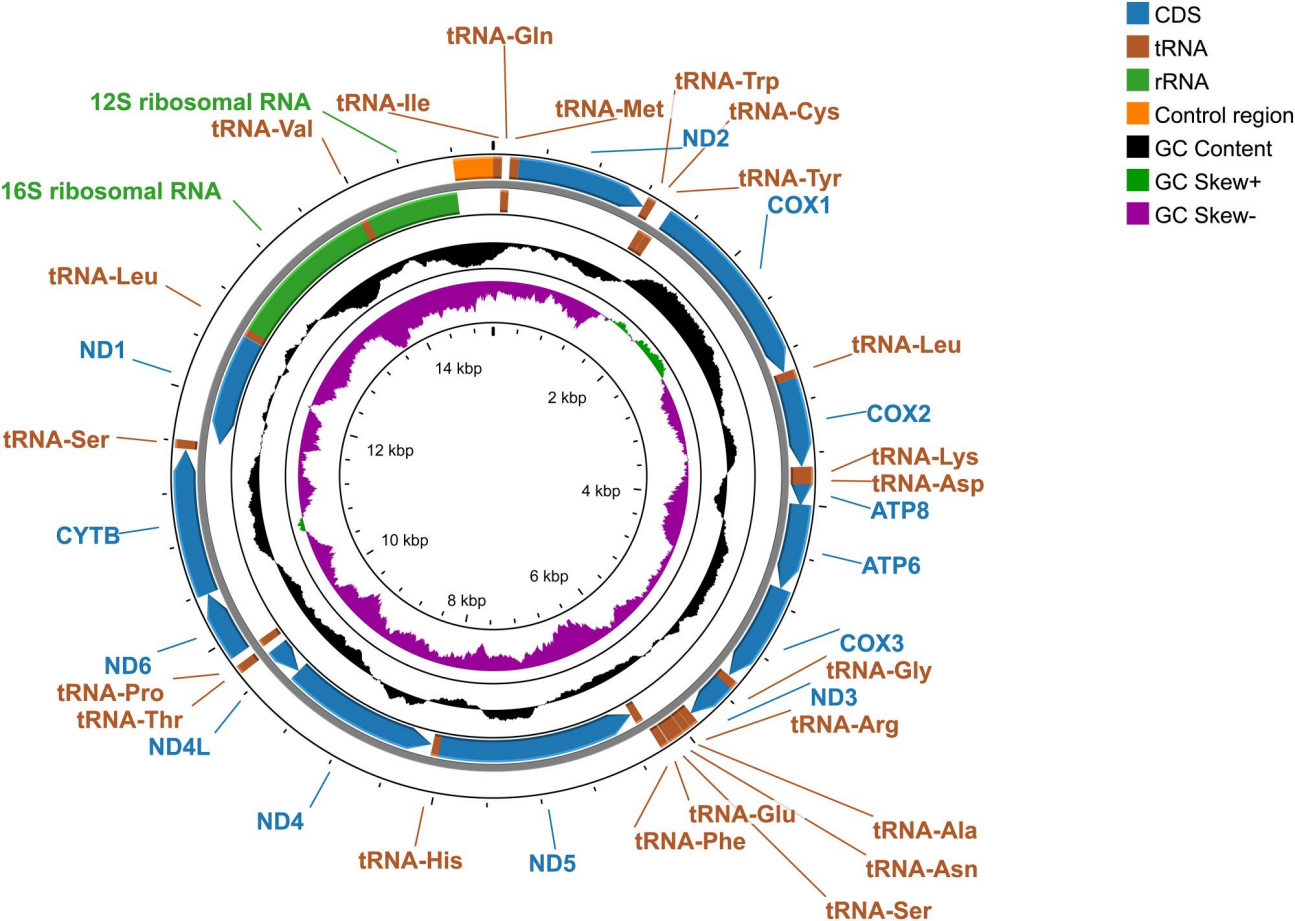
Gene map of the mitogenome of *E. semipurpurella alpina.*

The complete mitogenome of *E. semipurpurella alpina* is a circular DNA molecule with a length of 15,107 base pairs and contains 37 genes, including 13 protein-coding genes (PCGs), 22 tRNA genes, 2 rRNA genes, and one control region (Figure 2). Its sequence was deposited in GenBank under the accession number PP697947. The average coverage depth of the mitogenome was 1022.7× (Supplementary Figure S1). The nucleotide composition is 40.5% A, 40.1% T, 8.1% G, 11.3% C, exhibiting a positive AT skew (0.0042) and negative GC skew (−0.1683). The 13 PCGs start with typical ATN codons (ATT, ATG). *COX1*, *COX2* and *ND5* use a single T as an incomplete stop codon, the remaining PCGs terminate with canonical stop codons. The length of tRNA genes ranged from 62 to 71 bp. The 16S rRNA and 12S rRNA in this genome are 1,319 bp and 788 bp, respectively. The control region of *E. semipurpurella alpina* is 305 bp in length and harbors the highest A + T content (96.7%), compared to those in PCGs (79.0%), tRNAs (82.7%), and rRNAs (85.0%).

Circular maps were generated using Proksee (Grant et al. 2023). The outermost circle indicates the arrangement of genes: outer genes from the forward-strand; inner genes from the reverse-strand. GC skew was plotted, with positive skew in green and negative skew in purple, representing the deviation from the average GC skew of the entire sequence.

**Figure 3.**
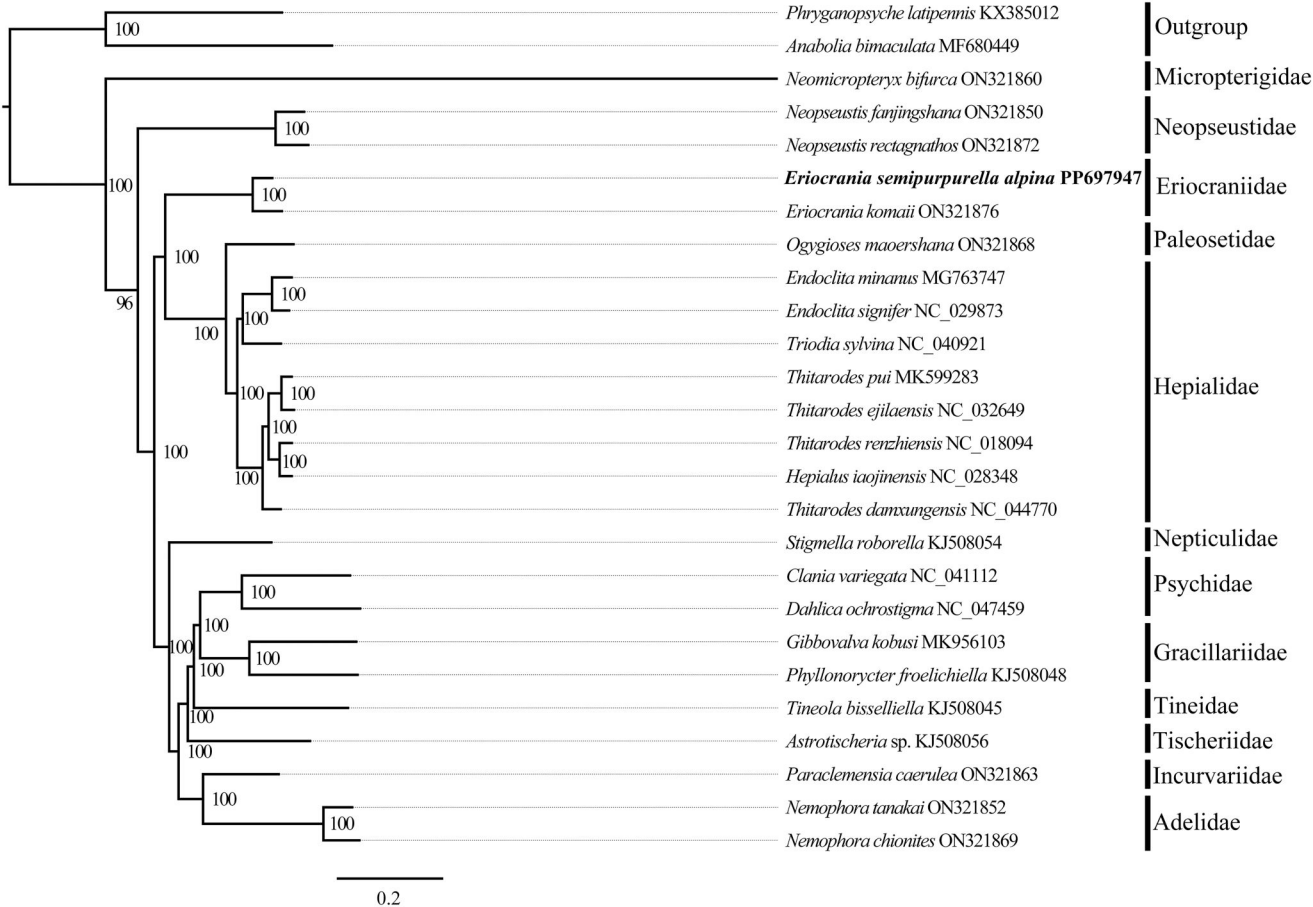
Mrbayes v3.2.7a (Ronquist et al. 2012) was used to construct the phylogenetic tree using 13 PCG sequences of *E. semipurpurella alpina* along with 23 species of lepidoptera and 2 species of trichoptera. Bayesian posterior probability values (%) are shown at nodes. GenBank accession numbers for the sequences are placed next to the species names. The newly sequenced species are highlighted in bold. The following sequences were used: *Phryganopsyche latipennis* (KX385012) (unpublished), *Anabolia bimaculata* (MF680449) (Peirson and Marcus 2017), *Neomicropteryx bifurca* (ON321860) (Liao et al. 2023), *Neopseustis fanjingshana* (ON321850) (Liao et al. 2023), *Neopseustis rectagnathos* (ON321872) (Liao et al. 2023), *Eriocrania komaii* (ON321876) (Liao et al. 2023), *Eriocrania semipurpurella alpina* (PP697947) (this study), *Ogygioses maoershana* (ON321868) (Liao et al. 2023), *Endoclita minanus* (MG763747) (unpublished), *Endoclita signifer* (NC 029873) (Yang et al. 2016), *Triodia sylvina* (NC 040921) (unpublished), *Thitarodes pui* (MK599283) (Zhang et al. 2019), *Thitarodes sejilaensis* (NC 032649) (Zhang et al. 2019), *Thitarodes renzhiensis* (NC 018094) (Cao et al. 2012), *Hepialus xiaojinensis* (NC 028348) (unpublished), *Thitarodes damxungensis* (NC 044770) (Zhang et al. 2019), *Clania variegata* (NC 041112) (Jeong et al. 2018), *Dahlica ochrostigma* (NC 047459) (Jeong et al. 2018), *Gibbovalva kobusi* (MK956103) (Chen et al. 2019), *Phyllonorycter froelichiella* (KJ508048) (Timmermans et al. 2014), *Tineola bisselliella* (KJ508045) (Timmermans et al. 2014), *Astrotischeria* sp (KJ508056) (Timmermans et al. 2014), *Paraclemensia caerulea* (ON321863) (Liao et al. 2023), *Nemophora tanakai* (ON321852) (Liao et al. 2023), *Nemophora chionites* (ON321869) (Liao et al. 2023), *Stigmella roborella* (KJ508054) (Timmermans et al. 2014).

The phylogenetic analysis revealed that *E. semipurpurella alpina* is the sister taxon to *Eriocrania komaii* (Figure 3). Additionally, Eriocraniidae is considered one of the most primitive families within the order Lepidoptera.

## Discussion and conclusion

In this study, we report the first complete mitogenome of *E. semipurpurella alpina* and provide a detailed annotation. The gene orientation and arrangement of this species are consistent with those of the sequenced species within the family Eriocraniidae (GenBank accession: ON321876). The family Eriocraniidae clade is supported by high Bayesian posterior probability values, grouping it with the families Hepialidae and Paleosetidae—a result consistent with previous research (Liao et al. 2023). To date, only two species within the family Eriocraniidae have been studied at the mitogenome level (Liao et al. 2023). Thus, further investigation is required based on additional molecular information.

## Supplementary Material

Figure S1.png

## Data Availability

The genome sequence data that support the findings of this study are openly available in GenBank of NCBI at https://www.ncbi.nlm.nih.gov/ under the accession No. PP697947. The associated BioProject, SRA, and Bio-Sample numbers are PRJNA1258583, SRR33524158, and SAMN48325431 respectively.
