# Supplementary figures and images for: The complete mitogenome of Eriocrania semipurpurella alpina Xu, 1990 (Lepidoptera: Eriocraniidae)
Source: Mitochondrial DNA B Resour. 2025 Dec 30;11(1):201–4. doi: 10.1080/23802359.2025.2609369 (PMC12777926; doi:10.1080/23802359.2025.2609369)

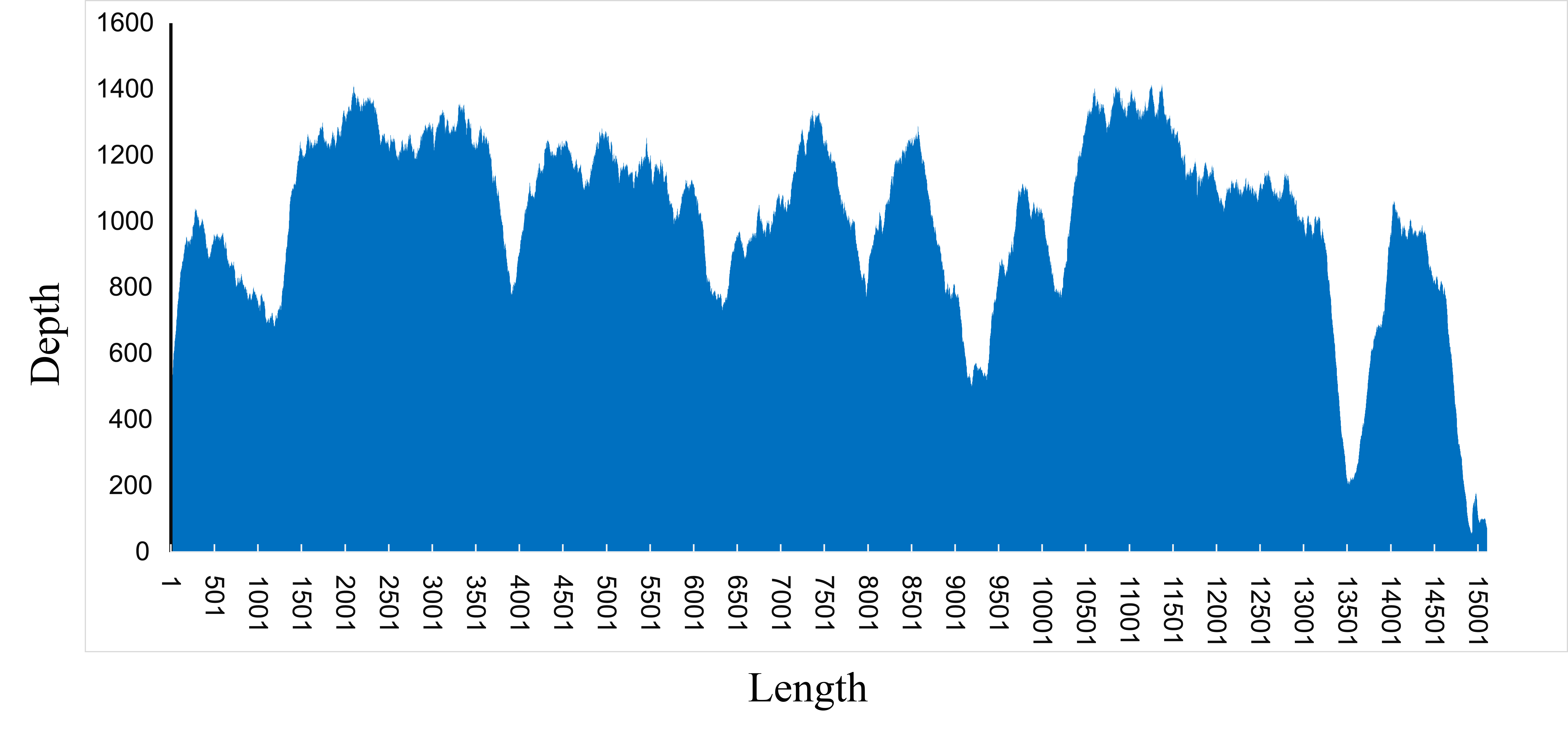

Supplement: Figure S1.png [file TMDN_A_2609369_SM9336.png]
